# Human umbilical cord mesenchymal stem cells therapy for Alzheimer’s disease: a systematic review and meta-analysis of mouse models

**DOI:** 10.3389/fneur.2026.1783757

**Published:** 2026-03-02

**Authors:** Chunyan Si, Liang Ma, Wei Ding, Yunxia Tian, Jianping Zhang, Hua Cao, Ya Shao, Zhiqiang Fan

**Affiliations:** Gansu Provincial Hospital of Traditional Chinese Medicine, Lanzhou, China

**Keywords:** Alzheimer’s disease, dose dependence, human umbilical cord mesenchymal stem cells, meta-analysis, transplantation route

## Abstract

**Objective:**

Given the limitations of current treatments for Alzheimer’s disease (AD), this study aims to comprehensively evaluate the therapeutic efficacy of human umbilical cord mesenchymal stem cells (hUCMSCs) in AD mouse models through a systematic review and meta-analysis. Additionally, we explore the impact of transplantation dose and route on treatment outcomes to identify the optimal window for clinical application.

**Methods:**

In accordance with the PRISMA guidelines, we systematically searched four major databases to identify randomized controlled trials involving hUCMSCs in AD mouse models. We used the standardized mean difference (SMD) to synthesize effect sizes and performed subgroup analyses based on pre-defined transplantation routes and doses.

**Results:**

A total of 13 studies were included in the analysis. The meta-analysis revealed that hUCMSCs transplantation significantly improved spatial learning and memory in AD model mice, with a marked reduction in escape latency (SMD = −2.55; 95% CI: −3.34 to −1.75; *I*^2^ = 77.9%, random-effects model). Additionally, it significantly lowered brain β-amyloid levels (SMD = −5.34; 95% CI: −7.21 to −3.47; *I*^2^ = 80.3%), increased brain-derived neurotrophic factor (SMD = 4.25; 95% CI: 3.18 to 5.31), and reduced neuronal apoptosis (SMD = −4.96; 95% CI: −6.52 to −3.41). Subgroup analyses further revealed that efficacy was significantly dose- and route-dependent. For cognitive improvement, intravenous injection of medium to high doses (≥1 × 10^6^ cells) was most effective and robust. For amyloid-β clearance, low-dose administration via intravenous, lateral ventricle, and cortical routes showed significant efficacy, whereas bilateral hippocampal injection did not yield significant benefits.

**Conclusion:**

Human umbilical cord mesenchymal stem cells can improve behavioral and pathological outcomes in AD mouse models via multiple mechanisms of action. The intravenous route using medium to high doses emerges as a critical factor for achieving optimal effects, providing important evidence and informing future experimental design and clinical translational research.

## 1 Introduction

Alzheimer’s disease (AD) is the leading cause of dementia, characterized by progressive cognitive and functional decline, often accompanied by a long-term need for care and a significant socioeconomic burden. Epidemiological data indicate that over 56 million people worldwide are currently living with dementia, a number expected to continue rising due to population aging. Consequently, the direct and indirect costs associated with dementia have become an unavoidable long-term strain on public health systems (1–3). However, existing mainstream pharmacological treatments, such as cholinesterase inhibitors and *N*-methyl-d-aspartate receptor antagonists, provide only limited symptomatic relief and are unable to halt or reverse the underlying pathological processes of the disease (1, 4, 5). In recent years, disease-modifying therapies such as monoclonal antibodies targeting amyloid-beta (Aβ) have made breakthroughs, but their heterogeneous efficacy, potential risk of imaging abnormalities, and high costs have limited their widespread application (5, 6). Therefore, the search for disruptive therapeutic strategies capable of multi-target intervention in AD’s complex pathological mechanisms, with the potential to achieve neurorepair, has become a critical challenge in the field.

Beyond cognitive impairment, AD is frequently accompanied by a range of neuropsychiatric symptoms (NPS), such as anxiety, depression, and apathy. Collectively, these manifestations are referred to as the behavioral and psychological symptoms of dementia (BPSD) and profoundly compromise the quality of life of both patients and their caregivers (7, 8). The hippocampus, a key brain region for cognition and emotional regulation, is a common pathological substrate underlying both cognitive deficits and BPSD in patients with AD (9).

In this context, mesenchymal stem cells (MSCs) have gained attention as promising therapeutic candidates due to their self-renewal capacity, multipotent differentiation potential, robust immunomodulatory functions, and ability to secrete neurotrophic factors (10, 11). Among the various sources of MSCs, human umbilical cord-derived mesenchymal stem cells (hUCMSCs) offer distinct advantages for clinical translation. Derived from perinatal medical waste, hUCMSCs are ethically uncontroversial, exhibit superior proliferative ability, lower immunogenicity, and notably potent paracrine activity (12, 13). Preclinical evidence from AD animal models also provides biological feasibility for the use of hUCMSCs. Previous studies in transgenic mouse models have shown correlations between hUCMSCs transplantation and reductions in Aβ load, as well as improvements in behavioral outcomes such as learning, memory, and sensorimotor functions, suggesting that hUCMSCs may exert a comprehensive effect by modulating the pathological microenvironment (13–16). Recently, Murayama (17) published a review that systematically summarized diverse therapeutic strategies targeting Aβ and tau proteins and highlighted the potential value of stem cell therapy in the treatment of AD, providing an important theoretical foundation for the present work. However, existing animal studies differ markedly in the types of models used, and the pathological characteristics and disease timelines vary across AD mouse models. For example, the APP/PS1 model co-expresses human APP carrying the Swedish mutation (K595N/M596L) and human PSEN1 carrying the L166P mutation, leading to increased Aβ42 production and progressive plaque deposition; the 5xFAD model co-expresses APP mutations (Swedish, Florida [I716V], and London [V717I]) together with PSEN1 mutations (M146L/L286V), resulting in extremely rapid pathological progression; whereas the Tg2576 model overexpresses human APP with the Swedish mutation alone, with later onset of Aβ deposition and cognitive deficits. In addition, some studies have used SAMP8 mice, which exhibit accelerated senescence, or models induced by hippocampal injection of Aβ oligomers (18, 19). This diversity in model types, together with substantial variation in the route, dose, and frequency of hUCMSC administration, may not only obscure the true magnitude of treatment effects but also make it difficult to translate seemingly consistent positive into reproducible clinical protocols.

Given this context, the present study aims to provide a more accurate estimate of the overall effect size of hUCMSCs on cognitive, behavioral, and core pathological outcomes through a rigorous and comprehensive systematic review and meta-analysis within a unified cell source framework. Subgroup analyses will be conducted to identify key variables that influence efficacy, such as administration routes, dose/frequency, and outcome measurement systems. The goal is to identify potential “optimal windows” for therapeutic intervention and, through systematic bias risk and reporting quality assessments, to provide a higher level of evidence for optimizing future animal study designs and clinical translation pathways.

## 2 Methods

### 2.1 Study design

This study was designed, conducted, and reported in strict adherence to the Preferred Reporting Items for Systematic Reviews and Meta-Analyses (PRISMA) guidelines.

### 2.2 Inclusion and exclusion criteria

#### 2.2.1 Study type

Only randomized controlled trials (RCTs) involving AD mouse models were included, regardless of whether the methods of allocation concealment or blinding were explicitly reported.

#### 2.2.2 Study subjects

Studies using any strain of AD mouse models (e.g., APP/PS1, 3xTg-AD, 5xFAD) or modeling methods (such as transgenic techniques, or Aβ oligomer injections) were included.

#### 2.2.3 Interventions

The experimental group must have undergone transplantation with hUCMSCs that were neither genetically modified nor induced to differentiate. The intervention dose and administration route of hUCMSCs had to be clearly reported. Control groups included blank controls (AD model without treatment), vehicle/sham surgery controls (e.g., PBS injections), and positive controls (e.g., different doses or routes of transplantation).

#### 2.2.4 Outcome measures

The included studies must report at least one of the following outcomes: (1) Escape Latency in the Morris Water Maze Test: Reflecting spatial learning and memory ability. The Morris water maze is the gold-standard assay for assessing hippocampus-dependent spatial learning and memory in rodents. It relies on the animals’ natural tendency to learn the location of a hidden platform in water, and quantifies spatial learning by recording the time from entry into the water to reaching the platform (i.e., escape latency). Shorter escape latency indicates more accurate memory of the platform location and better spatial learning and memory performance. Accordingly, improvement in escape latency is a core parameter for evaluating the efficacy of an intervention in restoring cognitive function (20). (2) Brain-Derived Neurotrophic Factor (BDNF) Expression: Either protein or mRNA levels, reflecting neuroprotection and synaptic plasticity. (3) Percentage of Apoptotic Cells: Typically assessed using methods such as TUNEL staining, to evaluate the extent of neuronal cell death. (4) Aβ Load: Including brain tissue Aβ plaque deposition area/quantity or Aβ1-42/Aβ1-40 levels.

#### 2.2.5 Exclusion criteria

Non-randomized studies, reviews, meta-analyses, conference abstracts, case reports, and *in vitro* studies were excluded. Studies using non-AD models, non-mouse species, or models with other severe neurological comorbidities were excluded. Studies whose full text or key data could not be accessed were excluded. Studies involving engineered, drug-pretreated, or bio-material composite hUCMSCs were excluded. Studies that did not report hUCMSCs source, transplantation dose, or route of administration were excluded, as were studies with severe data deficiencies or apparent inconsistencies.

### 2.3 Literature search strategy

We conducted a systematic search of PubMed, Embase, Web of Science, and Scopus databases from their inception up to November 1, 2025. The search strategy combined both subject headings and free terms, tailored to the indexing characteristics of each database. Intervention-related search terms included “human umbilical cord mesenchymal stem cells,” “umbilical cord-derived mesenchymal stem cells,” “hUC-MSCs,” etc.; disease-related terms included “Alzheimer disease,” “Alzheimer dementia,” “dementia,” etc. To minimize the risk of missing relevant studies, we also performed manual supplementary searches by tracing the reference lists of included studies and high-quality reviews in the field. Detailed search strategies and their adjustments for each database are provided in Supplementary Table 1.

### 2.4 Literature screening and data extraction

Literature screening was independently performed by two researchers with training in systematic review and animal study methodology. First, all search results were imported into reference management software, and duplicates were removed. Titles and abstracts were reviewed to exclude studies clearly unrelated to AD, mice, or hUCMSCs, or those that did not meet the predefined PICOS criteria. Full-text articles of the remaining studies were obtained for further screening, with a focus on verifying species, model type, interventions and controls, confirmation of human umbilical cord-derived MSCs, and reporting of the predefined outcome measures such as escape latency, BDNF, apoptosis, or Aβ levels. Disagreements on inclusion were resolved through discussion. If consensus could not be reached, a third senior researcher made the final decision. Reasons for exclusion were recorded, and a PRISMA flow diagram was created to present the entire screening process and the number of studies at each stage.

Data extraction was performed independently by two researchers using a pre-designed standardized electronic form, followed by cross-checking for accuracy. Extracted information included: Study details (first author, publication year, country). Animal and model characteristics (mouse strain, sex, age, weight, sample size per group, AD model type and induction method). Intervention and control details (cell dose, administration route, type and treatment of control groups). Outcome measure details. Methodological quality-related information was also recorded. For studies presenting results only in graphical format, values were extracted from graphs using GETDATA software. When multiple time points were reported in the same study, the most recent follow-up after the intervention was selected as the primary analysis time point.

### 2.5 Risk of bias assessment

The risk of bias for the included studies was evaluated using the SYRCLE Risk of Bias tool for animal studies. This tool, based on the Cochrane risk of bias framework, assesses multiple dimensions such as random sequence generation, baseline balance, allocation concealment, randomization of housing and environment, blinding of researchers and animal caregivers, blinding of outcome assessment, incomplete outcome data, selective reporting, and other potential biases. Two researchers independently performed the assessment, classifying each item as “low risk,” “high risk,” or “unclear risk.” Disagreements between the researchers were resolved through discussion or adjudication by a third senior researcher. The overall risk of bias for each item was displayed in a figure, along with the combined risk assessment.

### 2.6 Statistical analysis

All statistical analyses were performed using R software (version 4.4.2) and its meta package. Statistical tests were two-sided, with a significance level set at *p* < 0.05. All predefined outcomes were continuous variables. To account for differences in measurement methods, units, and scales across studies, standardized mean differences (SMD) and 95% confidence intervals (CI) were used as effect sizes. Heterogeneity between studies was assessed using Cochran’s *Q* test (with a significance level *α* = 0.10) and *I*^2^ statistics. Heterogeneity was considered low when *I*^2^ ≤ 50% and *Q* test *p* ≥ 0.10, in which case a fixed-effects model was used. Substantial heterogeneity (*I*^2^ > 50% or *p* < 0.10) warranted the use of a random-effects model (restricted maximum likelihood estimation of between-study variance *τ*^2^). For results with significant heterogeneity, clinical and methodological differences were considered, and subgroup or sensitivity analyses were conducted to explore potential sources.

Subgroup analyses were performed based on transplantation route and dose. Routes were categorized as intravenous or central (e.g., ventricular, hippocampal, or cortical injections). Doses were categorized as low (<1 × 10^6^ cells), medium (1–2 × 10^6^ cells), and high (≥2 × 10^6^ cells), with priority given to single-dose categorization. If only total doses were reported without specific dose differentiation, sensitivity analysis was performed to evaluate the impact of dose classification on the results. The consistency between fixed and random-effects models was compared to assess the robustness of the conclusions. Funnel plots were used to visually assess small sample effects and publication bias when the number of studies for a specific outcome was sufficient. Egger’s regression test and/or Begg’s rank correlation test were used for quantitative assessment, with *p* < 0.05 indicating potential publication bias. If significant publication bias was detected, the trim-and-fill method was used to adjust the pooled effect size, and differences between the adjusted and unadjusted results were compared to evaluate the potential impact of publication bias.

## 3 Results

### 3.1 Literature search results

**Figure 1:**
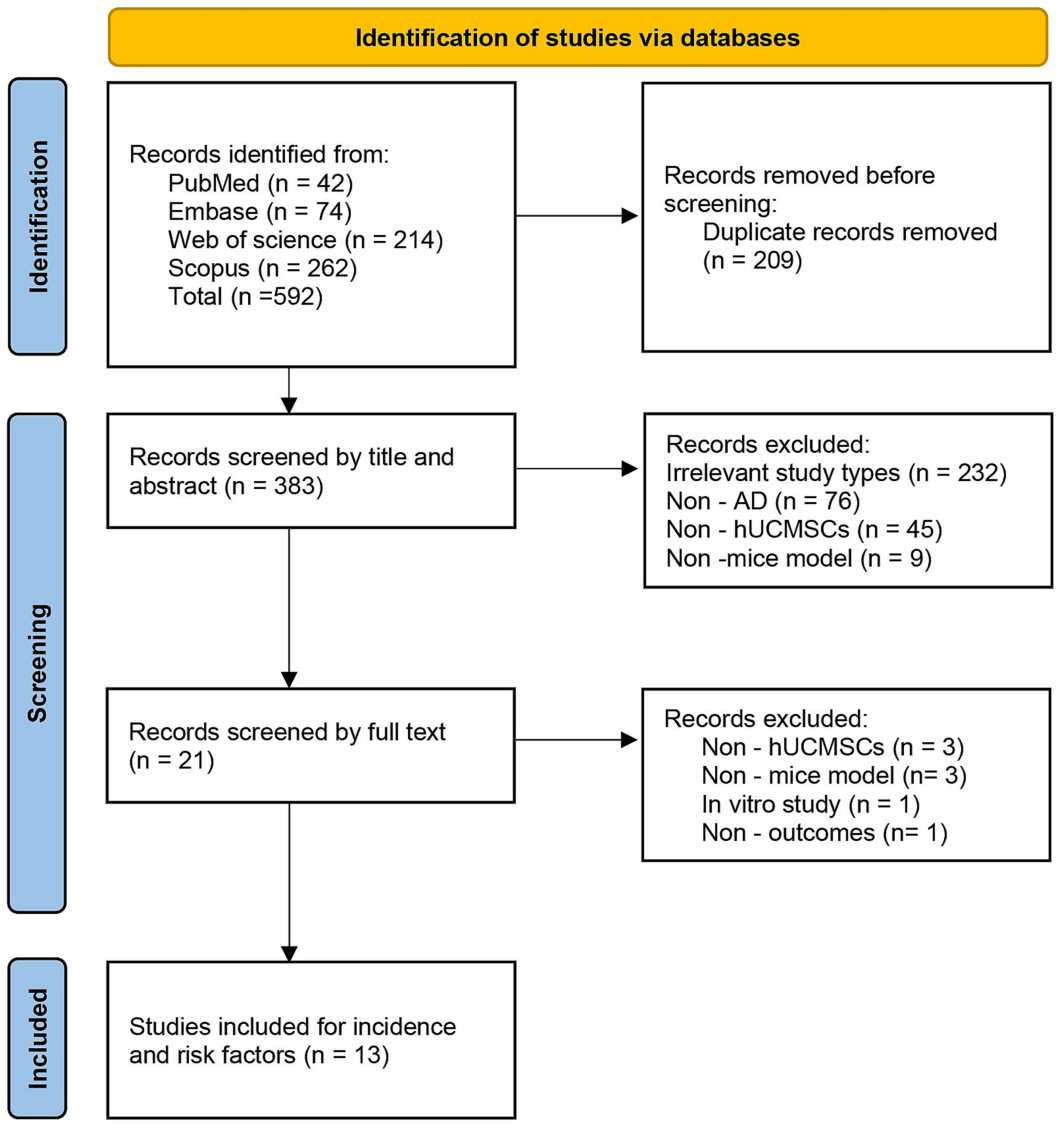
PRISMA flow diagram.

A total of 592 records were initially identified. After removing duplicates using EndNote software and manual checking, 383 unique records were retained for screening. Based on title and abstract review, 362 records were excluded as they were irrelevant to the study topic. The remaining 21 studies underwent full-text review and detailed evaluation. At this stage, 8 studies were excluded for not meeting the predefined inclusion criteria. Ultimately, 13 randomized controlled animal studies that met all inclusion and exclusion criteria were included in this analysis. The complete literature screening process is shown in Figure 1.

### 3.2 Basic characteristics of included studies

The 13 studies included in the final analysis were published between 2013 and 2024. All studies used AD mouse models. The most common model was transgenic mice (12 studies, 92.3%), including the APP/PS1 double transgenic model (6 studies), Tg2576 model (2 studies), 5xFAD model (2 studies), APPswe/PS1dE9 model (1 study), and SAMP8 accelerated aging model (1 study). One study used an Aβ₁–₄₂ hippocampal injection-induced AD model. Of the 9 studies that reported sex, all used male animals; ages ranged from 3 to 12 months. The intervention for all experimental groups involved the transplantation of non-genetically modified hUCMSCs. The transplantation routes varied, including peripheral routes (tail vein injection, 7 studies; intraperitoneal injection, 1 study) and central routes (hippocampal injection, 2 studies; intracerebroventricular injection, 2 studies; cortical injection, 1 study). The total transplant doses ranged from 0.1 × 10^6^ to 5 × 10^6^ cells. According to the predefined subgroup analysis criteria, the studies were categorized into low-dose (≤1 × 10^6^, 5 studies), medium-dose (1–2 × 10^6^, 5 studies), and high-dose (≥2 × 10^6^, 3 studies) groups. All control groups received either a vehicle control (phosphate-buffered saline [PBS] or saline) or a blank control. Detailed characteristics of each study are provided in Supplementary Table 2.

### 3.3 Risk of bias assessment results

**Figure 2:**
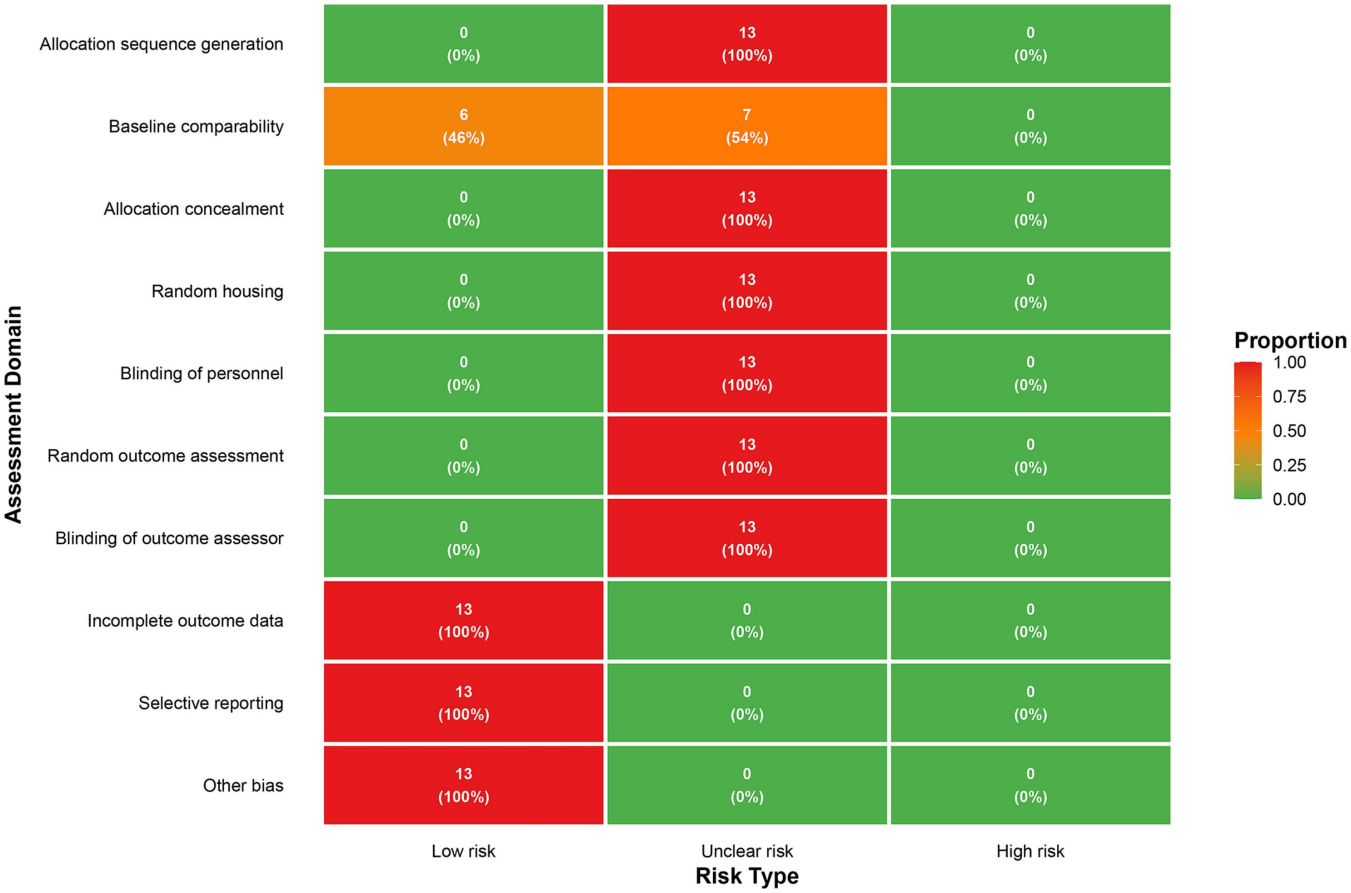
Risk of bias assessment results.

Several key deficiencies were identified in the reporting of experimental design and implementation, particularly regarding blinding and randomization details, which may introduce performance and detection biases. Specifically, all studies were rated as having an “unclear risk of bias” due to the lack of clear descriptions regarding random sequence generation, allocation concealment, random housing of animals during the experiment, blinding of animal caregivers and experimenters, and blinding of outcome assessors. In terms of baseline comparability, 6 studies (46.2%) reported comparable baseline characteristics between groups and were rated as “low risk,” while the remaining 7 studies (53.8%) were rated as having an “unclear risk” due to insufficient information. Regarding reporting-related biases, all studies fully reported all predefined outcome data with no missing data, and no other significant sources of bias were found. Consequently, they were rated as “low risk” for the following domains: “incomplete outcome data,” “selective outcome reporting,” and “other biases” (Figure 2).

### 3.4 Meta-analysis results

#### 3.4.1 Escape latency

**Figure 3:**
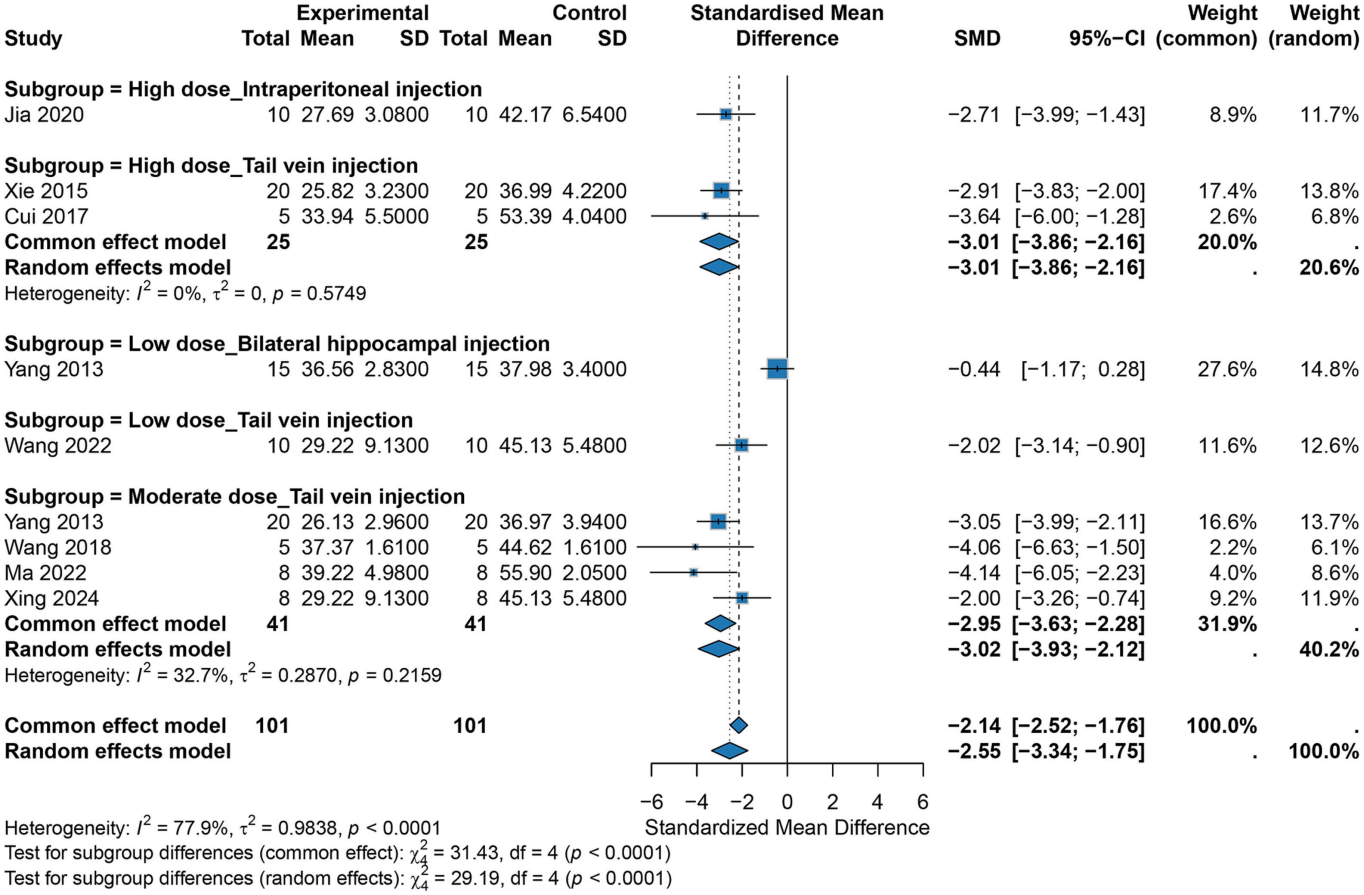
Meta-analysis results for escape latency.

A total of nine studies reported escape latency. The pooled analysis showed that hUCMSCs significantly reduced escape latency in AD mice (random-effects model: SMD = −2.55, 95% CI: −3.34 to −1.75), indicating an improvement in spatial learning and memory; however, there was substantial heterogeneity across studies (*I*^2^ = 77.9%). Further analysis revealed that both dose and route of administration were important determinants of efficacy. Notably, subgroup analyses stratified by dose and route substantially accounted for the observed heterogeneity. For tail vein injection, high and medium doses produced significant and comparable effects (SMD = −3.01 and −2.95, respectively), whereas the effect of the low dose was relatively weaker (SMD = −2.02). At the same high dose, tail vein injection yielded a slightly greater effect than intraperitoneal injection (SMD = −3.01 vs. -2.71). Under low-dose conditions, tail vein injection remained effective (SMD = −2.02), while intrahippocampal injection did not show a significant effect (SMD = −0.44, 95% CI: −1.17 to 0.28). Tests for subgroup differences confirmed statistically significant differences in effect sizes among dose and route subgroups (*p* < 0.0001) (Figure 3). In addition, heterogeneity within each subgroup was markedly reduced to an acceptable level (e.g., *I*^2^ = 0% in the medium-dose tail vein subgroup and I^2^ = 41% in the high-dose tail vein subgroup), confirming that our subgrouping strategy was both effective and appropriate.

#### 3.4.2 Amyloid β-protein (aβ)

**Figure 4:**
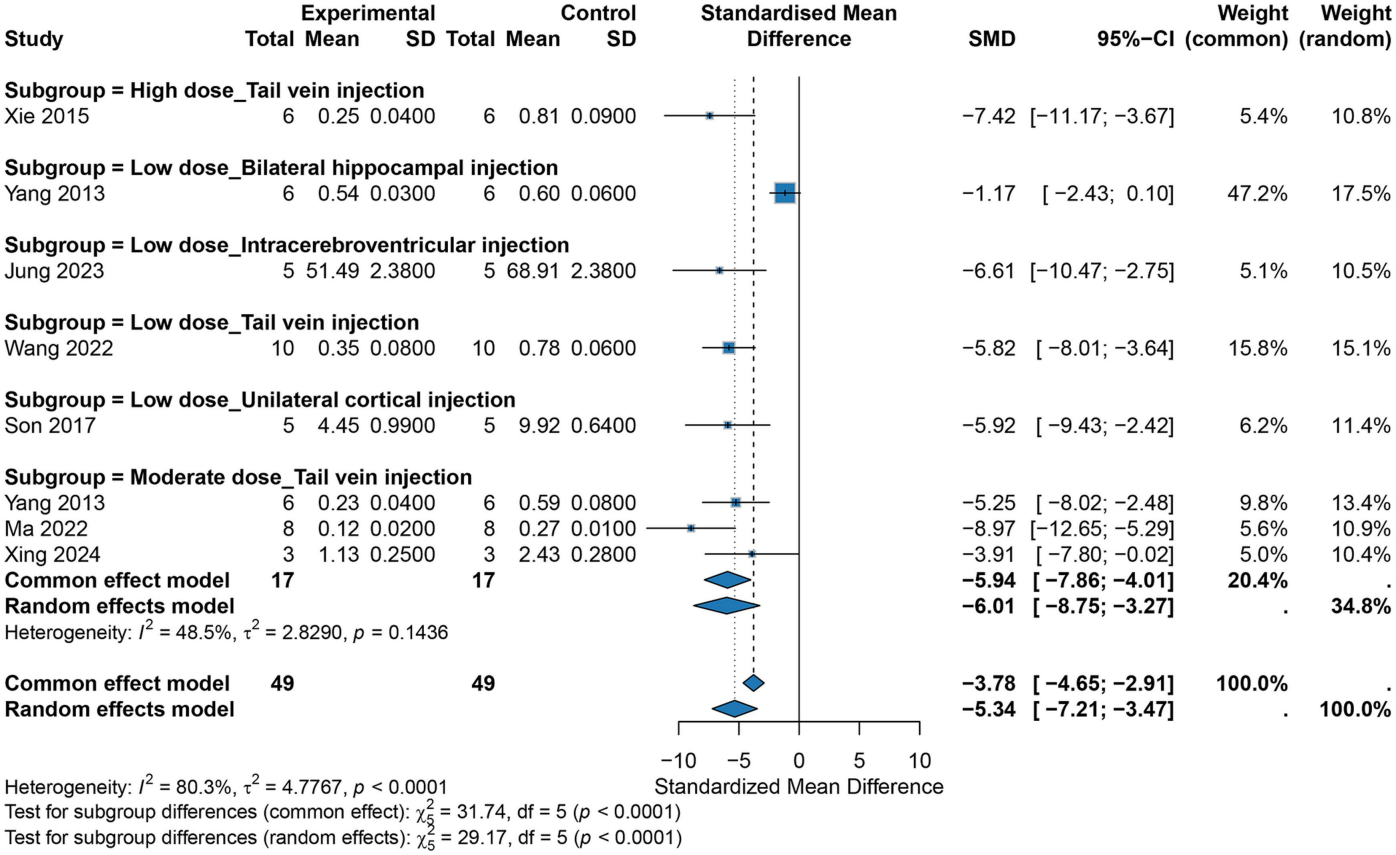
Meta-analysis results for Aβ.

Eight studies evaluated the effects of hUCMSCs on brain β-amyloid (Aβ) levels in AD mice. A random-effects meta-analysis showed that hUCMSCs treatment significantly reduced Aβ levels in model mice (SMD = −5.34, 95% CI: −7.21 to −3.47), indicating an amelioration of amyloid pathology; however, there was marked heterogeneity across studies (*I*^2^ = 80.3%, *p* < 0.0001). Subgroup analyses demonstrated that both dose and route of administration had a substantial impact on efficacy (between-subgroup *p* < 0.0001) and markedly reduced heterogeneity. At the same (low) dose, the effects of different administration routes differed considerably. Tail vein injection (SMD = −5.82), intracerebroventricular injection (SMD = −6.61), and unilateral cortical injection (SMD = −5.92) all significantly lowered Aβ levels, whereas intrahippocampal injection did not yield a significant effect (SMD = −1.17, 95% CI: −2.43 to 0.10). Within the tail vein subgroups, heterogeneity was reduced to 0% for all dose categories. When the route was held constant (tail vein injection), the effect size varied by dose. High (SMD = −7.42), medium (SMD = −5.94), and low (SMD = −5.82) doses all produced statistically significant effects, with an apparent dose-dependent pattern (Figure 4).

#### 3.4.3 BDNF

**Figure 5:**
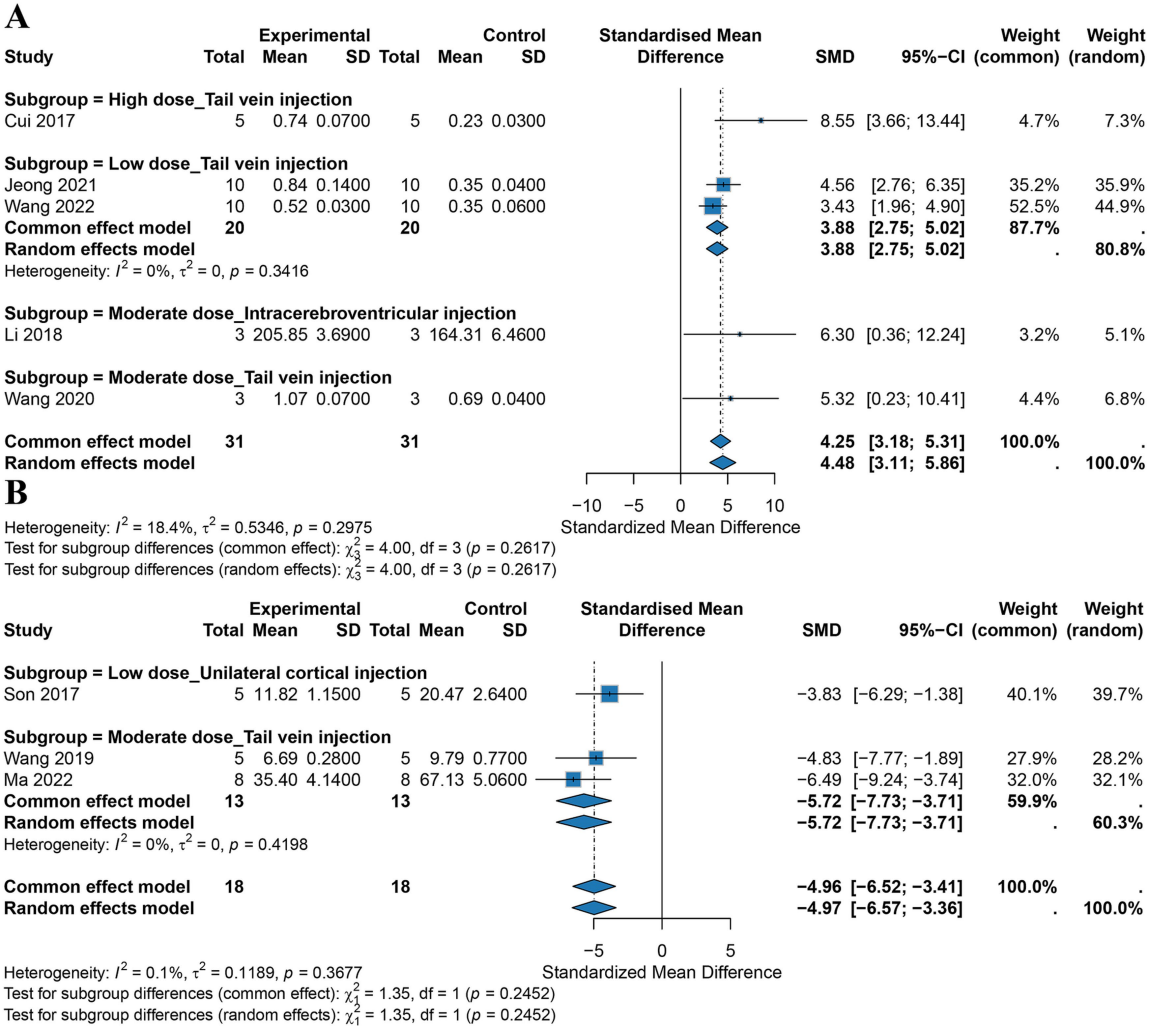
Meta-analysis results for BDNF and apoptosis levels (**A** BDNF; **B** apoptosis levels).

Four studies evaluated the effects of hUCMSCs on BDNF levels in AD mice. A fixed-effects model meta-analysis showed that stem cell treatment significantly increased BDNF levels in the brains of AD mice (SMD = 4.25, 95% CI: 3.18 to 5.31), suggesting a neurotrophic and neuroprotective effect, with low heterogeneity between studies (*I*^2^ = 18.4%, *p* = 0.2975). For the same administration route (tail vein injection), a dose-related trend was observed. The high-dose effect was most pronounced (SMD = 8.55), followed by the medium dose (SMD = 5.32), with the low dose showing a weaker but still significant effect (pooled SMD = 3.88), indicating a potential dose-dependent effect on BDNF enhancement when administered via tail vein. At the same dose level (medium dose), comparison of different administration routes showed that both tail vein injection (SMD = 5.32) and intracerebroventricular injection (SMD = 6.30) significantly increased BDNF levels, with similar effect sizes, suggesting that both routes at the medium dose are effective for upregulating neurotrophic factors. Subgroup analysis did not find significant between-subgroup differences across different dose and route combinations (*p* = 0.2617) (Figure 5A). Detailed results of the meta-analysis are presented in Table 1.

**Table 1:** Summary of meta-analysis results for hUCMSCs efficacy in different AD models and administration regimens.

Outcome	AD model type	Administration route	Dose (×10^6^)	No. of studies	Pooled SMD (95% CI)	*I* ^2^
Escape latency	Tg2576 transgenic mice; APPswe/PS1dE9 double transgenic mice	Tail vein	High dose (≥2)	2	−3.01 [−3.86, −2.16]	0%
	Tg2576 transgenic mice; APP/PS1 transgenic mice	Tail vein	Medium dose (1–2)	4	−3.02 [−3.93, −2.12]	32.70%
	SAMP8 (senescence-accelerated mouse prone 8)	Intraperitoneal	High dose (≥2)	1	−2.71 [−3.99, −1.43]	–
	APP/PS1 transgenic mice	Bilateral hippocampus	Low dose (<1)	1	−0.44 [−1.17, 0.28]	–
	APP/PS1 transgenic mice	Tail vein	Low dose (1–2)	1	−2.02 [−3.14, −0.90]	–
Aβ load	APPswe/PS1dE9 double transgenic mice	Tail vein	High dose (≥2)	1	−7.42 [−11.17, −3.67]	–
	APP/PS1 transgenic mice	Tail vein	Medium dose (1–2)	3	−5.94 [−7.86, −4.01]	48.50%
	APP/PS1 transgenic mice	Tail vein	Low dose (<1)	1	−5.82 [−8.01, −3.64]	–
	APP/PS1 transgenic mice	Bilateral hippocampus	Low dose (<1)	1	−1.17 [−2.43, 0.10]	–
	5xFAD transgenic mice	Intracerebroventricular	Low dose (<1)	1	−6.61 [−10.47, −2.75]	–
	5xFAD transgenic mice	Cortex	Low dose (<1)	1	−5.92 [−9.43, −2.42]	–
BDNF	Tg2576 transgenic mice	Tail vein	High dose (≥2)	1	8.55 [3.66, 13.44]	–
	Bilateral intrahippocampal Aβ₁–₄₂ injection; APP/PS1 transgenic mice	Tail vein	Low dose (<1)	2	3.88 [2.75, 5.02]	0%
	APP/PS1 transgenic mice	Tail vein	Medium dose (1–2)	1	5.32 [0.23, 10.41]	–
	APP/PS1 transgenic mice	Intracerebroventricular	Medium dose (1–2)	1	6.30 [0.36, 12.24]	–
Apoptosis	Tg2576 transgenic mice; APP/PS1 transgenic mice	Tail vein	Medium dose (1–2)	2	−5.72 [−7.73, −3.71]	0%
	5xFAD transgenic mice	Cortex	Low dose (<1)	1	−3.83 [−6.29, −1.38]	–

#### 3.4.4 Cell apoptosis

A meta-analysis of three studies evaluated cell apoptosis in brain tissue following hUCMSCs treatment. Overall analysis showed very low heterogeneity between studies (*I*^2^ = 0.1%, *p* = 0.368). Using a fixed-effects model, the results indicated that hUCMSCs treatment significantly reduced cell apoptosis in the brain tissue of AD mice compared to the control group (SMD = −4.96, 95% CI: −6.52 to −3.41). Subgroup analysis revealed that the anti-apoptotic effect was most pronounced in the medium-dose group administered via tail vein injection, with a pooled effect size of SMD = −5.72 (95% CI: −7.73 to −3.71). Low-dose unilateral cortical injection also significantly reduced apoptosis, although the effect size was smaller (SMD = −3.83, 95% CI: −6.29 to −1.38) (Figure 5B).

#### 3.4.5 Publication bias assessment

**Figure 6:**
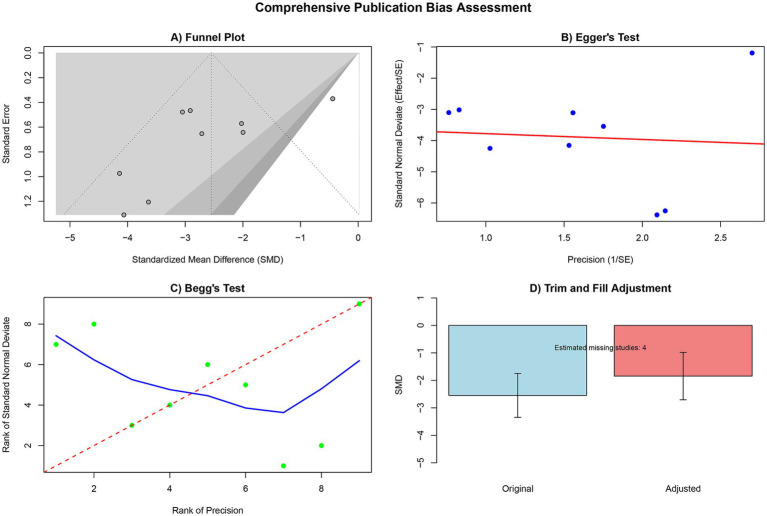
Publication bias detection results (**A** funnel plot; **B** Egger’s test; **C** Begg’s test; **D** trim and fill results).

A systematic assessment of publication bias was performed for the escape latency outcome. The funnel plot showed some degree of asymmetry, suggesting potential publication bias. Further quantitative tests, including Egger’s and Begg’s tests, confirmed the possibility of publication bias (*p* < 0.05). The trim-and-fill method estimated the potential existence of four missing studies. After adjustment, the pooled effect size was −1.85 (95% CI: −2.71 to −0.98), which differed from the original effect size (SMD = −2.55, 95% CI: −3.34 to −1.75), suggesting that the inclusion of these missing studies may affect the stability of the pooled results. However, the overall effect of hUCMSCs treatment remained significant (Figure 6).

## 4 Discussion

This study provides robust preclinical evidence for the use of hUCMSCs in treating AD mouse models through a rigorous systematic review and meta-analysis of 13 randomized controlled trials. Overall, our analysis not only confirms that hUCMSCs transplantation significantly reverses spatial learning and memory deficits in AD model mice but also reveals its dual role in alleviating core pathological features (Aβ load) and remodeling the neural microenvironment (upregulating BDNF and inhibiting apoptosis). Importantly, by exploring the sources of heterogeneity, we identified high-dose intravenous infusion as a potential optimal clinical translation window for achieving the best therapeutic effects, providing critical guidance for the design of future clinical trials.

Notably, Chen et al. (21) recently reported the first clinical observational study of intravenous infusion of human umbilical cord blood mononuclear cells (hUCBMCs) in patients with mild to moderate AD (21). In that study, 11 patients with AD were enrolled, and three infusions of hUCBMCs were found to transiently improve cognitive function (MMSE and ADAS-Cog scores), activities of daily living (ADL), and sleep quality (PSQI), with a favorable safety profile. Although hUCBMCs and hUCMSCs differ in cellular composition and biological properties (hUCBMCs comprise multiple cell populations, including hematopoietic and endothelial progenitors, whereas hUCMSCs are purified mesenchymal stem cells), both are umbilical cord-derived and share comparable immunomodulatory and neurotrophic potential. The clinical observations by Chen et al. (21) provide preliminary evidence for the feasibility and safety of umbilical cord-derived cell therapy in patients with AD, and are consistent with our meta-analytic findings, jointly supporting the therapeutic potential of umbilical cord-derived stem cells/cells for AD. Our systematic review further delineates the multi-target of hUCMSCs in animal models and suggests that medium-to-high dose intravenous infusion may be critical for optimizing clinical efficacy. These findings offer important guidance on dosing and administration routes for future clinical trials based on hUCMSCs.

Delving into the biological mechanisms, the cognitive benefits of hUCMSCs appear to stem from a multi-targeted, synergistic disease-modifying effect rather than a singular symptomatic treatment. Our meta-analysis results show a high degree of consistency between improvements in cognitive behavior and significant reductions in brain Aβ levels, along with a marked increase in BDNF protein expression. This suggests that hUCMSCs may exert neuroprotective and reparative effects through their paracrine activity. On one hand, the various bioactive factors they secrete can modulate the immune microenvironment in the brain, promoting the transformation of microglia into an anti-inflammatory phenotype (M2 type) with phagocytic capabilities, thereby enhancing the clearance of Aβ plaques (22, 23). On the other hand, hUCMSCs can continuously release neurotrophic factors such as BDNF, which not only support neuronal survival and synaptic plasticity but also directly counteract synaptic toxicity induced by Aβ oligomers and inhibit the resultant neuronal apoptosis (23–25). Compared to current clinical monoclonal antibody therapies that solely target Aβ clearance, this comprehensive intervention strategy, which both clears pathological products and actively promotes neural repair, clearly offers a more holistic advantage in addressing the complex pathological network of AD.

Regarding the choice of administration route, our study found that systemic administration (intravenous injection) demonstrated efficacy that was superior to or equivalent to that of local administration (hippocampal or ventricular injection) under specific conditions. Intuitively, direct injection into the brain should provide a higher local concentration of cells; however, our subgroup analysis revealed that bilateral hippocampal injection at low doses did not significantly improve cognitive behavior or reduce Aβ levels, while the same dose via tail vein injection showed significant efficacy. This seemingly paradoxical result may be attributed to local neuroinflammation caused by surgical trauma, which could offset the modest benefits of stem cells. It also suggests that hUCMSCs may possess a unique peripheral-central immune regulatory mechanism (26). At present, direct comparative evidence on different administration routes of hUCMSCs in AD models remains scarce, and some mechanistic insights are extrapolated from other disease models. For example, in ischemic stroke models, intravenously administered MSCs can confer neuroprotection by modulating peripheral immune cells (26). Building on these indirect data, we propose the following hypothesis for future testing: although most intravenously cells are initially trapped in the lungs, they may act by regulating peripheral immune organs such as the spleen and suppressing systemic inflammation, thereby improving the brain microenvironment through immune crosstalk along the gut–brain axis or across the blood–brain barrier (27–30). This raises the possibility that, for a disease such as AD, characterized by diffuse brain involvement and systemic metabolic disturbances, broad immunomodulation via the peripheral circulation may be more effective and safer than focal, target-specific injections. Nonetheless, this hypothesis requires validation in AD animal models through head-to-head comparisons of different administration routes and mechanistic experiments. Moreover, peripheral administration is also the most acceptable and readily translatable route for cell-based therapies in current clinical practice.

In addition to the administration route, dose dependence is another critical factor influencing therapeutic efficacy. Our data clearly demonstrate that under the tail vein administration route, the high-dose group exhibited significantly better Aβ clearance and BDNF enhancement compared to the low-dose group, indicating a pronounced “threshold effect” for hUCMSCs treatment. This suggests that a sufficient number of cells must be administered to generate a high enough concentration of cytokines to effectively reverse established pathological changes. This finding poses a challenge for human clinical translation, as simple allometric scaling based on body weight may result in a massive required cell quantity. Therefore, future translational research should focus on exploring how to maintain effective drug concentrations through multiple high-dose administration regimens in primate models while ensuring safety, thereby overcoming the efficacy threshold.

In addition to improving cognitive function, hUCMSCs transplantation may also exert beneficial effects on BPSD. Murayama et al. reported that neural stem cell transplantation significantly ameliorated anxiety- and depression-like behaviors in AD model mice, and that these improvements were associated with enhanced hippocampal neurogenesis and modulation of neurotransmitter systems such as the cholinergic and GABAergic pathways (31). Although the studies included in this meta-analysis primarily focused on cognitive performance and Aβ pathology, hUCMSCs may indirectly alleviate BPSD by reshaping the neural microenvironment, promoting the release of neurotrophic factors, and suppressing neuroinflammation. Future work should more systematically evaluate the impact of hUCMSCs on BPSD and incorporate these outcomes as an integral component of overall efficacy assessment.

Despite the encouraging evidence provided by this study, we must acknowledge that the included primary studies have notable methodological limitations. The risk-of-bias assessment showed that nearly all studies were judged as having an “unclear risk of bias” in key domains, including random sequence generation, allocation concealment, blinding of caretakers and investigators, and blinding of outcome assessment, suggesting a substantial likelihood of performance and detection bias at the level of the overall evidence base. Thus, although the meta-analysis indicates that hUCMSCs exert significant therapeutic effects in AD mouse models, the credibility of these findings is constrained by the generally low quality of the underlying studies. In addition, the effect sizes observed for outcomes such as Aβ reduction (SMD = −5.34) and BDNF elevation (SMD = 4.25) were extraordinarily large. Such inflated effect sizes are a common feature of preclinical meta-analyses and are typically closely linked to limitations in the original studies. As highlighted by the risk-of-bias assessment, blinding procedures were seldom clearly reported, sample sizes were generally small, and publication bias favoring positive findings likely contributed to systematic overestimation of treatment effects. For these pronounced pooled effect sizes, we therefore place greater emphasis on their direction rather than their absolute magnitude—that is, hUCMSCs treatment consistently favors improvement—rather than overinterpreting their possibly exaggerated numerical values. This interpretation is consistent with the observation that, after adjustment for publication bias, effect sizes decreased but remained significant, further supporting the robustness of the beneficial direction of hUCMSCs effects, while underscoring that the precise magnitude of benefit be confirmed in future large-scale, rigorously blinded, high-quality studies.

Of particular note, the impact of publication bias on the core outcomes cannot be ignored. Trim-and-fill adjustment for escape latency reduced the pooled effect size from −2.55 to −1.85, a decrease of approximately 27%. This substantial attenuation strongly suggests that, owing to missing negative or neutral studies, the observed pooled effect may be markedly overestimated and the true treatment effect is likely considerably smaller than the current pooled estimate. This further underscores that, when interpreting our findings, greater emphasis should be placed on the direction and robustness of the treatment effect rather than on its absolute magnitude. In addition, this study did not include several key AD-related outcomes such as tau pathology, synaptic markers, and neuroinflammation. These measures are central to AD pathogenesis and may represent important targets through which hUCMSCs exert their therapeutic actions. Because of the lack of available data in the primary studies, we were unable to quantify these effects, which limits a comprehensive understanding of the mechanisms underlying hUCMSCs therapy. Future animal experiments should systematically incorporate these endpoints to more fully evaluate the multi-target therapeutic potential of hUCMSCs.

Another critical limitation lies in the sex bias of the included studies. The nine studies that explicitly reported animal sex, all used male mice only. This pronounced sex bias substantially limits the generalizability of the findings. Given the well-established sexual dimorphism in AD—encompassing incidence, pathological features (such as patterns of Aβ deposition and tau phosphorylation), and immune–inflammatory responses—efficacy data derived solely from male animal models may not be readily extrapolated to females (32, 33). Of particular concern, the immunomodulatory properties of mesenchymal stem cells themselves may be influenced by sex-related hormonal and gene expression differences. Consequently, the current evidence base cannot address the pivotal question of whether hUCMSCs treatment confers comparable benefits in female AD models. Future preclinical studies must include both sexes to evaluate sex-specific treatment responses and to provide more broadly applicable evidence for dose selection and therapeutic regimens in subsequent clinical trials.

Second, a central limitation of this meta-analysis is the substantial heterogeneity observed in some pooled outcomes, particularly escape latency and Aβ levels. Although our prespecified subgroup analyses based on route and dose of administration successfully accounted for most of the heterogeneity and reduced within-subgroup heterogeneity to an acceptable range (with most *I*^2^ values < 50%), we cannot fully exclude the influence of additional confounders. These may include inherent differences in pathological mechanisms and disease trajectories across AD models (e.g., APP/PS1 vs. 5xFAD), as well as variation in mouse sex and age, length of post-transplant follow-up, and the specific analytical methods used. Such biological and methodological diversity is an inherent challenge in preclinical meta-analyses and may lead to overestimation or imprecise estimation of effect sizes. Consequently, while our subgroup findings provide important clues for identifying a potential optimal therapeutic window, they require confirmation in more standardized animal study designs. Based on the current evidence, we cannot assert the existence of a definitive “optimal clinical window,” but can only propose that medium-to-high doses administered intravenously represent a promising direction for further optimization. Future studies adhere closely to established guidelines for animal study design and reporting (such as the ARRIVE guidelines) to generate higher-quality evidence and provide a more robust foundation for clinical translation.

## 5 Conclusion

This study shows that hUCMSCs transplantation significantly improves spatial learning and memory deficits in AD mouse models, reduces brain β-amyloid burden, increases neurotrophic factor levels, and attenuates neuronal apoptosis. Subgroup analyses further suggest that the intravenous route and medium-to-high dosing may be key variables associated with greater efficacy. Although the low methodological quality of the primary studies (with a risk of effect inflation), the likelihood of publication bias, the pronounced sex bias limiting generalizability, and the residual heterogeneity in some outcomes all warrant caution, our findings nonetheless provide directional, systematic evidence supporting hUCMSCs as a promising disease-modifying therapy for AD and offer important guidance for optimizing treatment regimens in future clinical translation.

## Data Availability

The original contributions presented in the study are included in the article/Supplementary material, further inquiries can be directed to the corresponding author.
