# Supplementary material for: Human umbilical cord mesenchymal stem cells therapy for Alzheimer’s disease: a systematic review and meta-analysis of mouse models
Source: Front Neurol. 2026 Mar 2;17:1783757. doi: 10.3389/fneur.2026.1783757 (PMC12989406; doi:10.3389/fneur.2026.1783757)
Supplement: Supplementary file 1 [file Table_1.docx]

**Supplementary Table 1. Search strategy**

| 1.PubMed  #1: "Umbilical Cord"[MeSH Terms] AND "Mesenchymal Stem Cells"[MeSH Major Topic]  #2: "umbilical cord mesenchymal stem cells"[Title/Abstract] OR "umbilical cord derived mesenchymal stem cells"[Title/Abstract] OR "UC-MSC"[Title/Abstract] OR "umbilical cord mesenchymal stromal cells"[Title/Abstract] OR "wharton s jelly mesenchymal stem cell"[Title/Abstract]  #3: #1 AND #2  #4: "Alzheimer Disease"[MeSH Terms]  #5: "alzheimer s disease"[Title/Abstract] OR "alzheimer disease"[Title/Abstract] OR "cognitive impairment"[Title/Abstract] OR "dementia"[Title/Abstract]  #6: #4 or #5  #7: #3 and #6  2. WOS  (TS=(Umbilical cord mesenchymal stem cells OR Umbilical cord-derived mesenchymal stem cells OR UC-MSC OR Umbilical cord mesenchymal stromal cells OR Wharton's jelly mesenchymal stem cell)) AND TS=(Alzheimer’ s Disease OR Alzheimer Disease OR cognitive impairment OR dementia)  3. Scopus  TITLE-ABS-KEY ( "Alzheimer* disease" OR "Alzheimer disease" OR "Alzheimer&apos;s" OR "cognitive impairment" ) AND TITLE-ABS-KEY ( "Umbilical Cord" OR "Wharton&apos;s Jelly" OR "UC-MSC" ) AND ( LIMIT-TO ( DOCTYPE , "ar" ) OR LIMIT-TO ( DOCTYPE , "re" ) )  4. Embase  #1: 'umbilical cord mesenchymal stem cells':ti,ab,kw OR 'umbilical cord-derived mesenchymal stem cells':ti,ab,kw OR 'uc msc':ti,ab,kw OR 'umbilical cord mesenchymal stromal cells':ti,ab,kw OR 'whartons jelly mesenchymal stem cell':ti,ab,kw  #2: 'umbilical cord mesenchymal stem cells'/exp  #3: #1 OR #2  #4: 'alzheimers disease':ti,ab,kw OR 'alzheimer disease':ti,ab,kw OR 'cognitive impairment':ti,ab,kw OR dementia:ti,ab,kw  #5: 'alzheimer disease'/exp  #6: 'dementia'/exp  #7: #4 OR #5 OR #6  #8: #3 AND #7 |
| --- |

**Supplementary Table 2. Basic characteristics of the included studies**

| No. | Author | Year | Country | Research Type | Animal Characteristics | | | | Sample Size | Modeling Method | Stem Cells | | Transplantation Route | Transplantation Dose | Control Group |
| --- | --- | --- | --- | --- | --- | --- | --- | --- | --- | --- | --- | --- | --- | --- | --- |
|  |  |  |  |  | Species | Sex | Weight | Age | Experimental Group/Control Group |  | Type | Source |  |  |  |
| 1 | Cui | 2017 | China | RCT | C57BL/6 mice | Male | / | 12 months | 15/15 | Tg2576 transgenic mice | hUCMSCs | Human umbilical cord tissue | Tail vein injection | 2 × 10⁶ | PBS |
| 2 | Wang | 2018 | China | RCT | C57BL/6 mice | Male | 21.03 ± 1.7 g | 3 months | 15/15 | Tg2576 transgenic mice | hUCMSCs | Human umbilical cord tissue | Tail vein injection | 1× 10⁶ | Blank control |
| 4 | Son | 2017 | South Korea | RCT | 5xFAD transgenic mice | Male | / | 7 months | 5/5 | 5xFAD transgenic mice (expressing APP and PS1 mutations) | hUCMSCs | Human umbilical cord tissue | Unilateral cortical injection | 0.1 × 10⁶ | PBS |
| 5 | Xing | 2024 | China | RCT | Mice | Male | / | 6 months | 8/8 | APP/PS1 transgenic mice | hUCMSCs | Human umbilical cord tissue | Tail vein injection | 1 × 10⁶ | Saline |
| 6 | Wang | 2022 | China | RCT | Mice | / | / | 7 months | 10/10 | APP/PS1 transgenic mice | hUCMSCs | Human umbilical cord tissue | Tail vein injection | 0.2 × 10⁶ | PBS |
| 7 | Ma | 2022 | China | RCT | Mice | / | / | 6 months | 4/4 | APP/PS1 transgenic mice | hUCMSCs | Human umbilical cord tissue | Tail vein injection | 1 × 10⁶ | Saline |
| 8 | Jia | 2020 | China | RCT | Mice | Male | 30 g | 4 months | 10/10 | SAMP8 as a natural aging accelerated mouse model with AD-like pathology | hUCMSCs | Human umbilical cord tissue | Intraperitoneal injection | 5 × 10⁶ | PBS |
| 9 | Xie | 2015 | China | RCT | C57BL/6 mice | Male | / | 6 months | 20/20 | APPswe/PS1dE9 double transgenic mice | hUCMSCs | Human umbilical cord tissue | Tail vein injection | 2 × 10⁶ | PBS |
| 10 | Jeong | 2021 | South Korea | RCT | Mice | / | / | Adult | 10/10 | Bilateral hippocampal injection of Aβ₁–₄₂ | hUCMSCs | Human umbilical cord tissue | Tail vein injection | 0.5 × 10⁶ | Blank control |
| 11 | Jung | 2023 | South Korea | RCT | Mice | / | / | / | 10/10 | 5xFAD transgenic mice | hUCMSCs | Human umbilical cord tissue | Intracerebroventricular injection | 0.2 × 10⁶ | PBS |
| 12 | Yang | 2013 | China | RCT | Mice | Male | / | 6 months | 15/15 | APP/PS1 transgenic mice | hUCMSCs | Human umbilical cord tissue | Bilateral hippocampal injection | 0.1 × 10⁶ | PBS |
| 14 | Li | 2018 | China | RCT | Mice | Male | / | 6 months | 6/6 | APP/PS1 transgenic mice | hUCMSCs | Human umbilical cord tissue | Intracerebroventricular injection | 1 × 10⁶ | Saline |
| 15 | Yang | 2013 | China | RCT | C57BL/6 mice | Male | / | 6 months | 20/20 | APP/PS1 transgenic mice | hUCMSCs | Human umbilical cord tissue | Tail vein injection | 1 × 10⁶ | PBS |
